# Effective nutrition governance is correlated with better nutrition outcomes in Nepal

**DOI:** 10.1186/s12887-021-02898-4

**Published:** 2021-10-06

**Authors:** Grace Namirembe, Robin Shrestha, Julieta Mezzano, Lynne M. Ausman, Dale Davis, Kedar Baral, Shibani Ghosh, Gerald Shively, Patrick Webb

**Affiliations:** 1 Friedman School of Nutrition Science and Policy, Tufts University, 150 Harrison Avenue, Boston, MA 02111 USA; 2 Helen Keller International, Patan, Nepal; 3 Department of Community Health Sciences, Patan Academy of Health Sciences, Lalitpur, Nepal; 4 Department of Agricultural Economics, Purdue University, West Lafayette, IN USA

**Keywords:** Nutrition governance index, Policy processes, Stunting, Wasting, Nepal

## Abstract

**Background:**

The public health burden of undernutrition remains heavy and widespread, especially in low-income countries like Nepal. While predictors of undernutrition are well documented, few studies have examined the effects of political will and quality of policy or program implementation on child growth.

**Methods:**

Data were collected from two nationwide studies in Nepal to determine the relationship between a metric of nutrition ‘governance’ (the Nutrition Governance Index), derived from interviews with 520 government and non-government officials responsible for policy implementation and anthropometry measured for 6815 children in 5556 households. We employed Generalized Estimating Equation (GEE) and multilevel regression models.

**Results:**

A higher NGI (more effective nutrition governance) is positively associated with height-for-age as well as weight-for-height in children over 2 years of age compared to younger children (HAZ; *β* = 0.02, *p* < 0.004, WHZ; *β* = 0.01, *p* < 0.37). Results from the hierarchical model show that a one-point increase in the NGI is significantly associated with a 12% increase in HAZ and a 4% increase in WHZ in older children (> 24 months old). Mothers’ education, child’s age, BMI and no fever in the past 30 days were also protective of stunting and wasting. Seven percent and 17% of the overall variance in HAZ and WHZ, respectively, are accounted for by variations across the 21 district locations in which sampled households were located. Mean HAZ differs considerably across districts (intercept = 0.116, *p* < 0.001).

**Conclusions:**

These results highlight the importance of effective management of policy-based programming and resource use to bring about nutrition gains on the ground. The NGI explained a non-negligible amount of variation in HAZ and WHZ, which underscores the fundamental role that good governance plays in promoting child nutrition and growth, and the value of seeking to measure it to assist governments in moving policies from paper to practice.

## Background

The public health burden of undernutrition remains heavy and widespread, especially in low-income countries like Nepal, where rates are still very high, particularly in certain subgroups of the population [1]. The odds of stunting are four times higher in children born in low-income households compared to those from higher income households, which highlights inequalities not just in income, but also in access to, and use of health and nutrition services [2]. A recent anthropometric assessment found that 41% of pre-school children in the Mountains of Nepal were stunted compared with 19% of preschool children in the lower-elevation Terai (plains of Nepal), indicating significant geographic differences in the etiology of malnutrition. The prevalence of wasting in children under 5 years of age is estimated at 6.1% in the Mountains, 6.4% in the Hills, but 12.2% in the Terai [3]. A study by Shively et al., showed that “for each 1000 m gain in elevation, height-for-age Z-scores (HAZ) declined by between 0.1 and 0.2 points for an average child and by between 0.35 and 0.42 points for a child with the characteristics of those living at the highest elevations” [4]. The authors cited several factors beyond economic isolation as potentially contributing to the high stunting rates observed in the mountains, including differences in agricultural production and micronutrient deficiencies (zinc and iron). Poor sanitation and hygiene, and a low proportion of children receiving the minimum acceptable diet [5], are some of the factors associated with high wasting rates in the Terai relative to the other two regions.

Intermediate and underlying factors associated with stunting include the duration of breastfeeding, low size of baby at birth, low socio-economic status, economic isolation, and low and unpredictable rainfall. Other factors include seasonality, low maternal body mass index, lack of access to an improved water source, lower birth order, poor maternal education, and a high exposure to enteropathogens as evidenced by the levels measured in children’s stools [6–9]. Few studies have examined metrics of political will and quality of policy implementation relevant to nutrition, and their potential to support actions targeting factors associated with stunting. Given the enormous dependence of the public sector on health and nutrition services in Nepal, it is critical to elucidate whether the quality and intensity of government-supported actions aimed at improving nutrition play any measurable role in outcomes at the household level.

The concept of good governance (defined as “the effective implementation of national policies”) provides a platform for questioning the long menu of institutional changes and capacity building initiatives that are important and essential for development [10]. Good governance for nutrition entails four key elements including efficiency, accountability, transparency, and participation [11–13]. Most studies on nutrition governance have focused on evidence at a national or global level and the role of governance, focused on nutrition improvements, at the sub-national level has received limited attention. A critical gap remains in understanding the effectiveness of nutrition governance with a focus on the role of capacity, resources, information, coordination, and collaboration in achieving nutrition gains at the sub-national level [14].

This paper seeks to explore the association between the effectiveness of nutrition governance at national and subnational levels and key nutrition indicators such as stunting and wasting in Nepali children under 5 years of age. The paper is organized as follows: we start by presenting the study design for the two surveys used in this analysis. Then the Generalized Estimating Equation (GEE) model and multilevel model methods are described, before presenting results, including findings from regression analyses for both statistical approaches. We end the paper with discussion of the findings, articulation of study limitations, and recommendations for further research.

### Measuring the quality of nutrition governance

Measuring the quality of ‘governance’ is challenging. There is no consensus on a single theoretical definition of governance, thus it is difficult to determine what to measure. Yet there is need for governments to know if they are on track in improving governance systems in order to assess impact at the grassroots level and/or identfiy areas for improvement. To address this gap, several metrics have been proposed, including the World Governance Index [15] and the Hunger and Nutrition Commitment Index (HANCI) [16] among others, each with advantages and disadvantages. One study by Felismino et al. [17], explored types of national and sub-national governance (good versus not good) and their relationship with nutritional status of children in the Philippines. They collected data from focal point personnel in 30 municipalities and cities and created a single score out of four domains: 1) nutrition policies and programs, 2) organizational structure and resources for nutrition, 3) efficiency, and 4) accountability, transparency and participation. Cities and municipalities with a score above the median were defined as communities with “good governance”, that is, a governance structure supportive of nutrition. Furthermore, these good governance communities also had significantly lower underweight and stunting levels compared to those communities with lower scores (or poor governance structures). While these findings are in line with those of previous authors’ [18], the results obtained were based on bivariate analyses, thus were not robust because they did not account for possible confounding factors.

More recently, Namirembe et al. [19] developed a Nutrition Governance Index (NGI) that captures the effectiveness of nutrition governance at a sub-national level. The authors discussed at length the value of the NGI for uncovering relationships between the quality of governance and nutrition outcomes in children under five. This novel metric is an aggregate of five domains, as follows: i) understanding of nutrition and responsibilities by policy implementers, ii) collaboration, iii) financial resources, iv) capacity, and v) institutional support.

The domains used to create the NGI have been emphasized as important in facilitating improved nutrition outcomes [20–22]. For instance, the ‘knowledge’ domain has been found to be associated with height-for-age Z-scores (HAZ) in children under 5 years of age in Ghana [23]. Nutrition knowledge may arise from scholarly pursuits or community and societal engagements, supported by government and non-governmental initiatives. In Nepal, several initiatives provide nutrition knowledge as an intervention to improve nutrition. Examples of these include the USAID-funded SUAAHARA project and the Multi-sector Nutrition Plan (MSNP) [24], which sought to improve mother’s infant and young child feeding knowledge and behaviors, provide nutritional counseling and raise awareness regarding better health and nutrition practices, among other objectives to fight malnutrition in Nepal. The SUAAHARA project was initiated in twenty districts of Nepal from 2011 to 2016. The goal of the project was to improve nutrition, maternal, newborn, and child health services, reproductive health or family planning services, water, sanitation and hygiene, and home-based gardening. The MSNP project spanned from 2013 to 2017 with a similar aim of improving nutrition outcomes. Some of the objectives included setting action points for implementing nutrition-sensitive policies and strategies that encompass agriculture and public health, and reducing stunting in children and undernutrition in women [25].

The importance of technical knowledge for nutrition is clear. However, the scope of such interventions needs to be widened to include the relevant local leadership that is tasked with enforcing adherence to, and implementation of, protocols. Regarding the importance of collaboration, support and commitment, various authors have studied the role of inclusiveness of multiple professions on health outcomes, highlighting their importance in encouraging workers to speak up and participate in problem-solving and advancing shared goals [26–28]. These interventions can contribute to changes in children’s nutrition outcomes so we found it necessary to adjust for their influence, especially because there is an overlap between the time of their implementation and our study duration.

## Methods

### Study design and data collection

Data used here are derived from two separate but related studies: the Policy and Science for Health, Agriculture and Nutrition (PoSHAN) Community study, and the PoSHAN Policy study. The PoSHAN Community study was a nationally representative household panel study that was conducted annually from 2013 to 2016 in Village Development Communities (VDCs) across the three agroecological zones of Nepal (Mountains, Hills and Terai) [29]. At the time of the surveys, VDCs were the lowest administrative units in Nepal. The PoSHAN Policy study utilized the same study design and timeframe but focused on institutional respondents within VDCs. Thus, the PoSHAN Policy study sampled government and non-government officials while the PoSHAN Community study surveyed households, and women and children within these households. The PoSHAN Policy study targeted relevant offices and organizations within VDCs based on their defined responsibilities in implementing Nepal’s Multisector Nutrition Plan (MSNP). The MSNP is a collaborative multi-national partnership spearheaded by the government of Nepal to improve maternal and child nutrition and reduce chronic malnutrition, largely through evidence-based nutrition interventions [30].

Additional details regarding the PoSHAN Community study have been published elsewhere [29]. Briefly, seven VDCs were sampled from the three agroecological regions of Nepal (Mountains, Hills and Terai), resulting in twenty-one VDCs. From each VDC, three wards (roughly equivalent to neighborhoods) were selected at random. Households eligible for inclusion in the PoSHAN Community study were those with a child younger than 5 years or with a woman who was married within the past 2 years. The 2016 survey of the PoSHAN Community study included 5556 households from 64 wards, and 6815 children in total. The 2014 PoSHAN Policy study collected data at the ward level and included 523 participants. Questionnaires for both the POSHAN Community study and the POSHAN Policy study are publicly available [31]. The Tufts University Social Behavioral and Educational Research Board (SBER) and the Nepal Health Research Council (NHRC) provided human subjects research ethical approval.

To construct the dataset for this analysis, we matched the 2014 Policy data to the 2016 PoSHAN Community data by VDC. Although the Policy study sampled the same wards as the Community study, the selection of participants was not representative of the wards within the Community study. To correct for this imbalance, we aggregated NGI scores to the VDC level, which is the smallest administrative and geographic unit available for matching the two studies.

### Statistical analysis

The statistical analysis focuses on two child anthropometric measures: height-for-age Z-score (HAZ) and weight-for-height Z-score (WHZ). These nutrition indicators were calculated using WHO’s age- and sex-specific growth standard references [32]. We utilized Generalized Estimating Equation (GEE) models and Multilevel (also known as hierarchical) regression models for the multi-variate analyses. Both approaches provide advantages over conventional regression models in the context of our study design, where errors and outcomes for units are clustered within sample frames. Conventional regression models may underestimate the standard error of the mean and easily reject the null hypothesis because they do not account for this study design effect. In this situation a GEE model provides robust estimates of the variances of regression coefficients [33] and provides other benefits [34]. A multilevel regression approach provides the advantage of estimating variances at each level (in this case the child and the VDC), which avoids over-weighting realizations of the NGI that are repeated for all children within a VDC. All estimates were adjusted for distance to the market, child’s age, sex, diet diversity [35], fever in the past 30 days, mother’s education, mother’s BMI (Body Mass Index), maternal age, Food Insecurity and Access Scale [36], MSNP and SUAAHARA districts.

All analyses were conducted in SAS v 9.4. Both models satisfied tests for normality and absence of multicollinearity among regressors. Unless otherwise specified, associations are reported as statistically significant based on *p* < 0.05.

### Generalized estimating equation (GEE) models

The Generalized Estimating Equation model (GEE) is an extension of generalized linear regression models (GLM). As estimated, the GEE models included as explanatory variables those known from past studies to be associated with stunting and wasting in Nepalese children under 5 years of age [8, 37]. Child-specific variables included age, sex, diet diversity, report of any fever in the past 30 days, breastfeeding status, and whether the child received healthcare. Age squared was included to account for any non-linearity in the relationship between age and outcome. Mother-specific variables included duration of formal schooling, BMI and age. Household-specific food insecurity scores were also included as direct measures as well as indirect controls for household wealth.

### Multilevel regression models

**Fig. 1:**
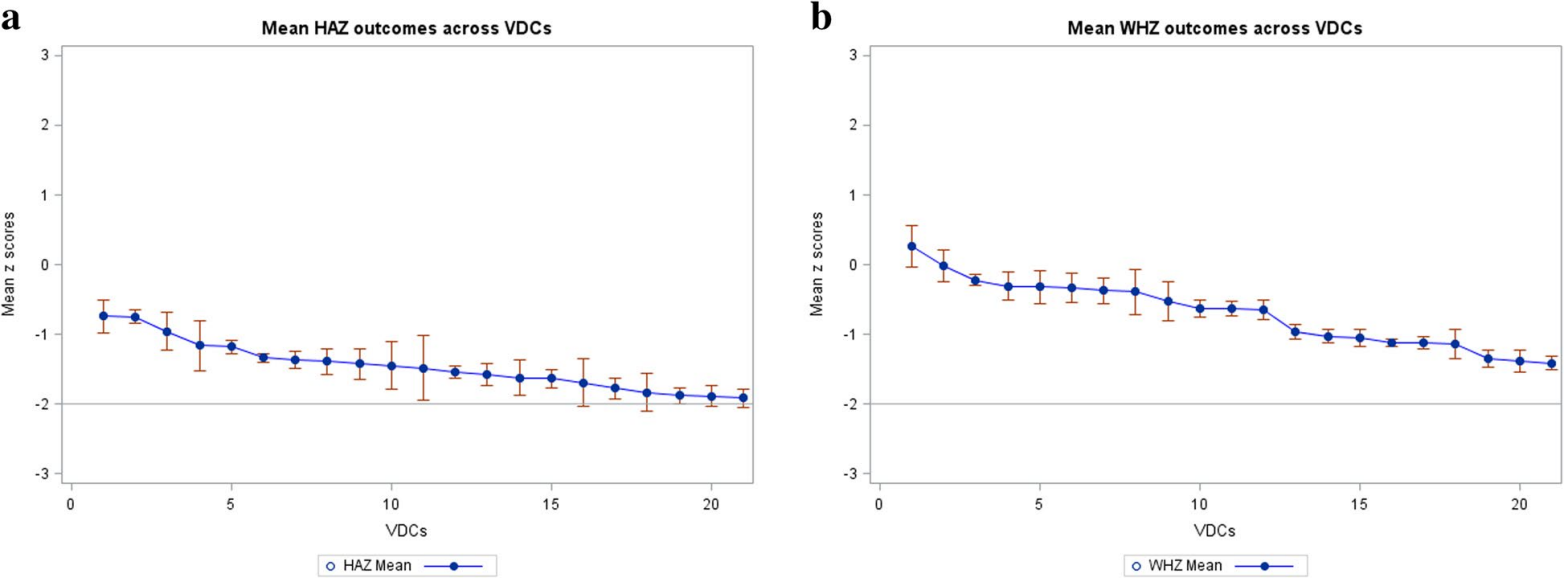
**a** Variations in mean HAZ across VDCs, **b** Variations in mean WHZ across VDCs

The multilevel models were fitted using the maximum-likelihood estimation method, allowing intercepts to vary across VDCs. To judge model fit, we relied on the Akaike Information Criterion (AIC). Lower values imply better fit [38]. In the multilevel regression, children are nested within VDCs. Accordingly, child outcomes constitute the primary level of analysis and the VDC constitutes the second level. We began analysis with a null model without predictors to determine whether mean HAZ and WHZ differed significantly across the 21 VDCs. The multilevel model further tests the alternative hypothesis that VDC-level predictors are correlated with HAZ and WHZ. We tested this hypothesis using the intra cluster correlation (ICC) value. The ICC indicates the extent to which children in the same VDC are similar based on their HAZ and WHZ relative to the total variation in HAZ and WHZ measured across all VDCs. If the within-cluster variance is high, then the resulting ICC is expected to be low. Koo et al. [39] states that an ICC less than 0.5 is poor. Although a multilevel model approach may not be ideal if the ICC indicates clustering by VDC is not substantial, this approach allows us to investigate VDC-level effects (NGI) on child-level outcomes (HAZ and WHZ). Figure 1a and b illustrate the variances in mean HAZ and WHZ across VDCs.

Successive models included child-level predictors and VDC-level predictors respectively. These models control for the nested design of the data by permitting VDC intercepts to vary while estimating fixed effects.

The Null model equation is:

1 $$Y_{ij}=\,\beta_{\mathit0j}+\,r_{ij}$$

2 $$\beta_{\mathit0j}=\Upsilon_{\mathit{00}}+\mu_{\mathit0j}$$

Substituting Eq. 2 into Eq. 1 results in estimating the Null equation:

3 $$Y_{ij}=\Upsilon_{\mathit{00}}+\mu_{\mathit0j\,+}r_{ij}$$

where

*Y*_*ij*_* is the HAZ or WHZ* outcome for child *i* within VDC *j*

*β*_*0*j_ is the average HAZ or WHZ for each VDC

*ϒ*_*00*_ is the grand mean of VDC intercepts

*µ*_*0j*_ is the variance of the intercepts

*r*_*ij*_ is the within-group variance in the outcome for each VDC

The successive random intercept and fixed slope, and interaction equations are denoted by:

4 $$Y_{ij}=\Upsilon_{\mathit{00}}+\Upsilon_{\mathit{10}}X_{ij}+\Upsilon_{\mathit{01}}W_j+\mu_{\mathit0j}+r_{ij}$$

5 $$Y_{ij}=\Upsilon_{\mathit{00}}+\Upsilon_{\mathit{01}}W_j+\Upsilon_{\mathit{10}}X_{ij}+\Upsilon_{\mathit{11}}X_{ij}W_j+\mu_{\mathit0j}+\mu_{\mathit1j}X_{ij}+r_{ij}$$

where

*Y*_*ij*_* is the HAZ or WHZ* outcome for child *i* within VDC *j*

*ϒ*_*00*_ is the grand mean of the outcomes across VDCs and children

*ϒ*_*10*_ is the regression coefficient associated with *X*_*ij*_

*ϒ*_*01*_ is the regression coefficient associated with W_j_

*ϒ*_*11*_ is the regression coefficient associated with the interaction term *X*_*ij*_*W*_*j*_

*W*_*j*_ is the VDC-level predictor (NGI)

*µ*_*0j*_ is the variance of the intercepts

*µ*_*1j*_ is the variance of the slopes

*r*_*ij*_ is the within-group variance in the outcome for each VDC

## Results

### Sample characteristics

The sample characteristics for women and infants participating in this study are presented in Table 1. The average Nutrition Governance Index (NGI) score was 52.36 (95% CL: 50.21–54.50) in 2014. The median distance to any market within VDCs was 0.52 miles. Regarding women’s characteristics, the average age was 27 years and the mean amount of time spent in school was 3.61 years (95% CL: 2.60–4.61) in 2014. Forty-seven percent of children were female. The average child’s age was 35.60 months in 2014. Average child’s weight was 11.20 kg (95% CL: 10.97–11.43). The prevalence of stunting and wasting was estimated at 34.0% (95% CL: 33–35%) and 14.0% (95% CL: 13–15%) respectively. Seventy-two percent of the households were food secure in 2014; slightly more households were food secure in 2016 (79.10%).

**Table 1:** Sample characteristics (*N* = 6815)

**Variable**	**2014 (Panel 2)**						**2016 (Panel 4)**					
	**N**	**Mean / %**	**95% CL**		**Min**	**Max**	**N**	**Mean / %**	**95% CL**		**Min**	**Max**
NGI	6815	52.36	50.21	54.50	43.54	62.68	7051	52.25	49.90	54.60	43.54	62.68
Market distance^a^	6815	0.52	0.22	0.81	0.02	2.07	7051	0.52	0.21	0.83	0.02	2.07
**Women’s characteristics**												
Education (years)	5947	3.61	2.60	4.61	0.00	17.00	6255	4.40	3.21	5.58	0.00	18.00
BMI	5943	20.60	19.98	21.21	13.10	47.01	6245	21.16	20.42	21.90	13.71	38.59
Age (years)	5953	27.42	26.68	28.16	10.00	76.00	6258	27.34	26.71	27.97	13.00	80.00
**Children’s characteristics**												
Sex												
*Female*	3212	47.13	-	-	-	-	3318	47.07	-	-	-	-
*Male*	3603	52.87	-	-	-	-	3731	52.93	-	-	-	-
Weight (kg)	6383	11.20	10.97	11.43	1.70	30.00	6661	11.46	11.23	11.68	1.50	24.00
Age (months)	6803	35.60	34.73	36.46	0.00	72.00	7032	36.89	35.88	37.90	0.00	75.00
Age categories												
≤ *24 months*	2345	34.47	-	-	-	-	2298	32.68	-	-	-	-
> *24 months*	4458	65.53	-	-	-	-	4734	67.32	-	-	-	-
HAZ	6342	-1.54	-1.57	-1.51	-5.97	5.75	6608	-1.44	-1.47	-1.41	-5.96	4.52
WHZ	5481	-1.01	-1.04	-0.99	-4.89	3.94	5565	-0.91	-0.93	-0.88	-4.98	3.72
Stunted	6344	37.11	35.92	38.29	-	-	6610	33.98	32.84	35.12	-	-
Wasted	5481	16.29	15.31	17.27	-	-	5565	13.60	12.70	14.50	-	-
CDDS^b^	6418	4.63	4.39	4.87	0.00	7.00	6706	4.86	4.67	5.06	0.00	7.00
Child visited health facility (no. of times)	6363	3.38	2.56	4.20	0.00	20.00	6600	3.49	3.02	3.95	0.00	22.00
No fever^d^												
*High*	829	12.92	-	-	-	-	1188	17.72	-	-	-	-
*Low*	1864	29.04	-	-	-	-	2199	32.80	-	-	-	-
HFIAS^c^												
*Food secure*	4634	72.24	-	-	-	-	5303	79.10	-	-	-	-
*Mild food insecurity*	959	14.95	-	-	-	-	606	9.04	-	-	-	-
*Moderate food insecurity*	612	9.54	-	-	-	-	540	8.05	-	-	-	-
*Severe food insecurity*	210	3.27	-	-	-	-	255	3.80	-	-	-	-

^a^Median distance to markets within VDCs

^b^Child’s diet diversity based on 7-day food frequency recall [31]

^c^Household Food Insecurity and Access Scale

^d^Child had no fever in the past 30 days

NGI was positively associated with HAZ and WHZ in older children (> 2 years old) compared to younger children (HAZ; *β* = 0.02, *p* < 0.004, WHZ; *β* = 0.01, *p* < 0.37) as seen in Table 2. Mothers’ education, her age and BMI, and absence of fever in the child during the past 30 days were all positively associated with HAZ and WHZ. Distance to the market and child’s diet diversity were negatively associated with HAZ and WHZ.

**Table 2:** Linear relationship between anthropometric indicators and NGI in Nepalese children

**Parameter**	**HAZ**		**WHZ**	
	**β (SE)**	***p* value**	**β (SE)**	***p* value**
NGI	-0.03 (0.02)	0.049	-0.04 (0.02)	0.03
Market distance^b^	-0.12 (0.13)	0.35	-0.33 (0.07)	< 0.001
**Children’s characteristics**				
Age (months)				
> *24*	-1.84 (0.46)	< 0.001	-0.38 (0.58)	0.51
≤ *24*	*Ref*	*Ref*	*Ref*	*Ref*
Age & NGI				
*NGI &* > *24*	0.02 (0.01)	0.01	0.01 (0.01)	0.38
*NGI &* ≤ *24*	*Ref*	*Ref*	*Ref*	*Ref*
Sex				
*Female*	-0.02 (0.03)	0.50	0.02 (0.03)	0.34
Month of birth	-0.01 (0.00)	0.00	-0.00 (0.01)	0.44
CDDS^a^	-0.15 (0.01)	< 0.001	-0.01 (0.01)	0.17
No fever^d^	0.03 (0.03)	0.33	0.15 (0.02)	< 0.001
**Women’s characteristics**				
Education	0.04 (0.01)	< 0.001	0.02 (0.00)	< 0.001
BMI	0.04 (0.01)	< 0.001	0.06 (0.01)	< 0.001
Age (years)	0.01 (0.00)	0.03	0.00 (0.00)	0.87
HFIAS^c^				
*Mild food insecurity*	0.32 (0.12)	0.01	0.09 (0.05)	0.05
*Moderate food insecurity*	0.28 (0.10)	0.00	0.17 (0.07)	0.02
*Food secure*	0.35 (0.10)	< 0.001	0.12 (0.05)	0.01
*Severe food insecurity*	*Ref*	*Ref*	*Ref*	*Ref*
MSNP Suaahara	0.05 (0.13)	0.69	0.12 (0.14)	0.39
Intercept	-0.07 (0.88)	0.94	0.10 (0.97)	0.92
**N**	6094		5127	

^a^Child’s diet diversity [31]

^b^Median distance to markets within VDCs

^c^Household Food Insecurity and Access Scale

^d^Child had no fever in past 30 days

The NGI score was aggregated from its five constituent domains. Estimates for its interaction with child’s age are presented in Table 3. Collaboration, financial resources and support domains were significantly and positively associated with HAZ in older children as was the knowledge domain for WHZ. We observed an unexpected negative and significant relationship between the nutrition knowledge domain with HAZ and support domain with WHZ in children over 2 years old.

**Table 3:** Relationship between NGI domains and HAZ and WHZ in older children (> 24 months old)

**NGI domain**	**HAZ**		**WHZ**	
	**β (SE)**^**a**^	***p* value**	**β (SE)**^**a**^	***p* value**
Nutrition knowledge	-0.02 (0.01)	0.02	0.03 (0.01)	< 0.001
Collaboration	0.01 (0.00)	0.01	-0.00 (0.00)	0.68
Financial resources	0.01 (0.00)	0.02	-0.00 (0.00)	0.24
Capacity	0.00 (0.00)	0.97	0.01 (0.00)	0.14
Support	0.04 (0.01)	< 0.001	-0.04 (0.02)	0.01
**N**	6094		5127	

^a^Beta estimates for the interaction between NGI domains and children’s age categories. Estimates are adjusted for NGI, market distance, child’s age, sex, month of birth, CDDS, fever and maternal characteristics (maternal education, BMI, age), HFIAS, MSNP and SUAAHARA districts

Results from the multilevel models are shown in Table 4. Because HAZ variation across VDCs was small (Fig. 1a and b) and the VDC-level analysis must be based on a small set of observations (21 VDCs), we investigated VDC-level effects using only one community-level variable, NGI. Interpretation of results was restricted to Model 4 for both outcomes as it was the best-fit or most improved model based on possessing the smallest AIC estimate. This means that adjusting for NGI together with its interaction with age, improved model fit for both outcomes.

**Table 4:** Multi-level model results for the relationship between height-for-age Z-score and NGI in Nepalese children

**Variable**	**HAZ**						**WHZ**							
	**Model 1**	**Model 2**		**Model 3**	**Model 4**		**Model 1**			**Model 2**	**Model 3**		**Model 4**	
**Child-level estimates**														
Intercept	-1.49***	-1.70***		-1.64***	-1.68***		-0.75***			-1.92***	-1.86***			-1.96***
Child’s age														
> *24 months*		-1.51***		-1.52***	-1.51***					0.14***	0.14***			0.14***
≤ *24 months*		*Ref*		*Ref*	*Ref*					*Ref*	*Ref*			*Ref*
Female child		-0.02		-0.02	-0.02					0.03	0.03			0.03
CDDS^a^		-0.14***		-0.14***	-0.14***					-0.02***	-0.02***			-0.02***
No fever^b^		0.04**		0.05**	0.05**					0.13***	0.14***			0.14***
Month of birth		-0.01**		-0.01**	-0.01**					-0.00	-0.00			-0.00
Mother’s education		0.04***		0.04***	0.04***					0.01***	0.01***			0.01***
Mother’s BMI		0.04***		0.04***	0.04***					0.06***	0.06***			0.06***
Mother’s age		0.01***		0.01***	0.01***					-0.00*	-0.00*			-0.00*
**Community-level estimates**														
NGI (Z-score)				-0.02		-0.09						-0.05	-0.07	
NGI (Z-score) & child’s age														
*NGI* & > *24 months*						0.12***							0.04*	
*NGI* & ≤ *24 months*						*Ref*							*Ref*	
Panel 2				-0.07**		-0.07***						-0.07***	-0.07***	
Panel 4				*Ref*		*Ref*						*Ref*	*Ref*	
**Covariance parameters**														
Intercept	0.116***		0.094***	0.094**		0.094**		0.199***	0.117**			0.115**	0.115**	
Residual	1.555***		1.367***	1.365***		1.362***		0.950***	0.902***			0.901***	0.901***	
ICC	0.07		0.06	0.06		0.06		0.17	0.11			0.11	0.11	
**Model fit statistics**														
**AIC**	42,546.8		37,608.4	37,601.8		37,574.4		30,878.9	27,854.5			27,843.0	27,841.4	
**N**	12,950		11,910	11,910		11,910		11,046	10,148			10,148	10,148	

^*^p < 0.10; ** *p* < 0.05; *** *p* < 0.001

^a^Child’s diet diversity score; ^b^Child had no fever in the past 30 days; Estimates are beta coefficients; Values based on SAS PROC MIXED; Estimation method is Maximum Likelihood

From Model 1, we calculated a significant ICC of 0.07 (95% CI, 0.04, 0.12) for HAZ and an ICC of 0.17 (95% CI, 0.10, 0.28) for WHZ. This indicates that 7 and 17% of the overall variances in HAZ and WHZ were accounted for by variations across VDCs. When the NGI was zero, the average estimated HAZ for all VDCs was -1.49 and the average estimated WHZ was -0.75. The covariance estimates for both the intercept and residual were significantly different from zero. This implies that average HAZ differed considerably across VDCs (intercept = 0.116, *p* < 0.001) and there was more variation in HAZ among children within VDCs (residual = 1.555, *p* < 0.0001) as reflected in the low ICC estimate.

**Fig. 2:**
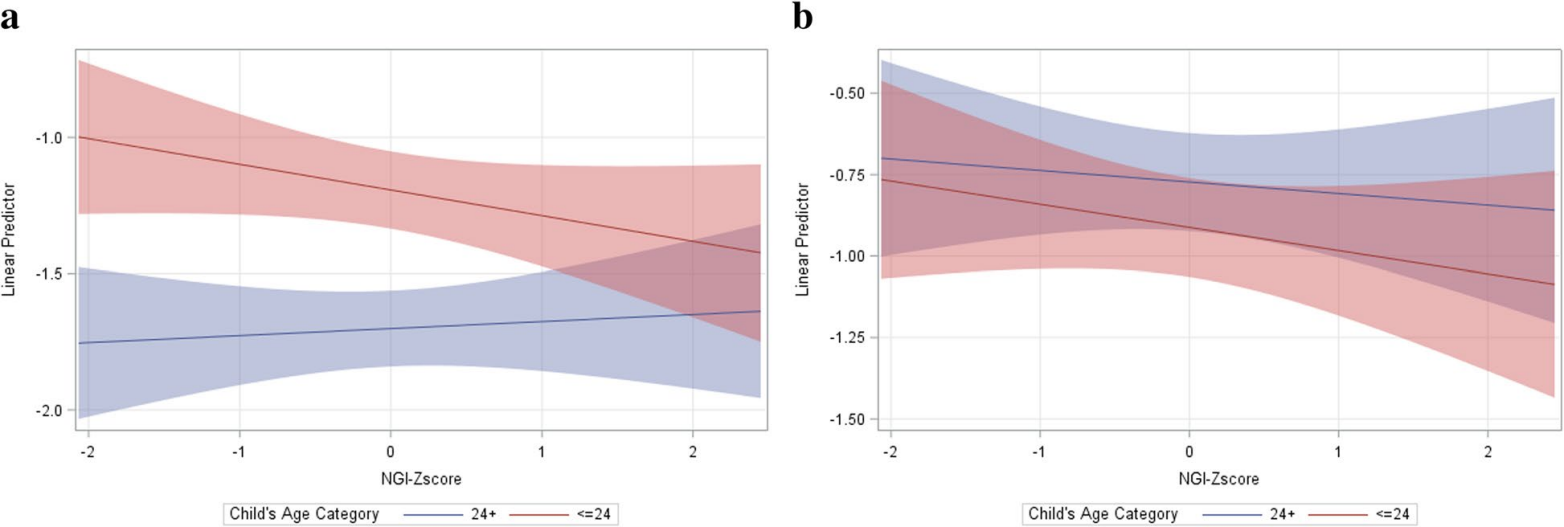
**a** HAZ and NGI by child’s age-group, **b** WHZ and NGI by child’s age-group

The positive and significant relationship between NGI and HAZ in older children, that was also observed in the GEE model, was observed across both outcomes (Fig. 2a and b). That is, a one-point increase in the NGI was significantly associated with a 12% higher average HAZ and a 4% higher WHZ in older children (> 24 months old). No reported fever, maternal education and maternal BMI were all positively associated with HAZ and WHZ.

The covariance parameters for the intercept in Model 4 were unchanged compared to previous conditional models for the same outcome. This minimal decrease in variance meant that NGI and its cross-level interaction with age, explained only a small portion of the between-VDC variations in HAZ and WHZ. Specifically, 19% (0.116–0.094/0.116) of the explainable variation in mean HAZ and 42% (0.199–0.115/0.199) of the explainable variation in WHZ were due to the NGI and its cross-level interaction respectively.

## Discussion

This paper measured the association between the effectiveness of nutrition governance and nutrition outcomes using a Nutrition Governance Index. After adjusting for other known predictors of stunting and wasting, we found that this relationship was positive for children over 2 years of age. This may reflect that policy actions rolled out in Nepal improve nutrition over time via multisectoral nutrition-sensitive pathways that typically benefit older children. More targeted nutrition-specific interventions are largely designed to affect pregnancy and post-pregnancy outcomes, usually delivered through health services alone. It may also reflect the lagged effects of changes in trainings or practice that may enhance the effectiveness of implementation over time. Since the NGI captured the effectiveness of nutrition governance at societal and community levels, it could reflect aggregate gains at those levels rather than shorter-term gains accruing specifically to mothers and infants. It has been shown that agriculture, education and infrastructure investments most likely support improvements in older children in Nepal who are already consuming the family diet as compared to babies and infants [40].

The results presented here underscore the point that factors associated with the child’s nutritional status are complex, ranging from individual to the community level. Individual and community-level variables explained 19% of the variation in HAZ and 42% in WHZ. Child-level variables were strong predictors of both outcomes and their effects persisted throughout the series of models that adjusted for higher-level variables, highlighting their importance in addressing malnutrition. This finding echoed findings in Bangladesh and Nigeria that showed the predominance of these factors in predicting stunting and wasting in children [41, 42]. Smith and Shively reported significant but relatively smaller overall contributions from community-level factors, although their investigation focused on district-level factors and did not address governance directly. They found that approximately 6% of total variance and 22% of explained variance in height-for-age Z-scores occurred between districts [43].

That between-VDC variance was relatively low in our null model reflects the fact that since Nepal’s VDCs are larger geographic units than wards, individual variability within these groups can be expected to be higher compared to smaller units. That said, Smith and Shively found that adding additional ward-level variables to a multilevel model of HAZ did not increase the explained proportion of second-level variance substantially [43].

We found a negative relationship between HAZ in older children and the ‘knowledge about nutrition’ domain of the NGI, which is surprising but an indication that there is need to improve nutrition knowledge acquisition. Our findings were in line with Saaka [23] who found a significant association between HAZ and maternal childcare knowledge. The counterintuitive direction for the magnitude of effects for the NGI domains on both nutrition outcomes could be attributed to two possible explanations: the deficits of using a cross-sectional approach and a lack of specificity in the nutrition knowledge domain. Cunningham et al. [44] studied predictors of changes in nutritional outcomes in Nepal from 1996 to 2011 and found that “health services were the single largest contributor to LAZ change,” with an estimated increase of 0.15 in LAZ over a 15-year period. This suggested that the impacts attributed to good governance, of which access to health services is a crucial component, occured after a long period. It is possible that our analyses, which were limited to a 2-year period, were not able to capture any measurable effects. The knowledge domain at its core measured an understanding of nutrition (nutrition problems and strategies to address any issues) and personal responsibilities in their roles as relevant officials that are key to interpreting and implementing nutrition policies. A negative relationship with HAZ may imply that this domain is not specific enough to capture the true effect of nutrition knowledge that may include knowledge of nutritious diets, agricultural and WASH practices that have been established as important factors associated with stunting. The negative relationship between the Support domain and WHZ was surprising and should be investigated further.

One limitation of this study was the reliance on one second-level variable to represent community-level predictors due to a small sample of VDCs and minor observed variation in nutrition outcomes between VDCs. However, when we estimated models using other community-level variables, such as distance to market, estimates and interpretations remained similar and model fit statistics did not improve. In addition, the Policy Process study recruited various office holders as participants in the study. Over time, some of these individuals changed offices or departments. Because the study design focused on the position, rather than the individual, the NGI may not have captured long-established governance practices, rendering its association with nutrition outcomes inaccurate.

Although our findings are based on two time points, the design used is multi cross-sectional so we cannot account for temporal trends that are likely to affect nutrition outcomes. However, the two statistical approaches used were appropriate in seeking to understand factors associated with stunting and wasting in Nepal. These results may not be generalizable to other contexts, as the questionnaire was adapted for Nepal although some of the questions can cut across all nutrition-relevant offices.

Magnusson et al. [45] revealed that assuming independence of errors associated with baseline indicators used in the creation of the World Governance Indicator [15] resulted in an inaccurate indicator. The NGI was modeled with the same assumption about measurement errors. We therefore recommend further investigation into alternative methodologies that relax this assumption and a reassessment of NGI in relation to nutrition outcomes.

## Conclusions

In Nepal, through an innovative set of analyses, we found that better nutrition governance was positively associated with nutrition outcomes (HAZ and WHZ) in children over 2 years of age. This result was obtained using two robust statistical approaches. Our findings underscore the importance not just of targeted interventions, but also of effective management of those interventions in the context of translating policies into practice. The multilevel approach revealed that NGI explained a non-negligible amount of variation in HAZ and WHZ, which underscores the fundamental role that good governance plays in promoting child nutrition and growth.

## Data Availability

The datasets used and/or analyzed during the current study are available from the corresponding author on reasonable request.

## Abbreviations

AIC: Akaike Information Criterion
BMI: Body mass index
GEE: Generalized Estimating Equation
GLM: Generalized linear regression models
HANCI: Hunger and Nutrition Commitment Index
HAZ: Height-for-age Z-scores
ICC: Intra cluster correlation
MSNP: Multisector Nutrition Plan
NGI: Nutrition Governance Index
NHRC: Nepal Health Research Council
PoSHAN: Policy and Science for Health, Agriculture and Nutrition
SBER: Social Behavioral and Educational Research Board
USAID: United States Agency for International Development
VDC: Village Development Communities
WHO: World Health Organization
WHZ: Weight-for-Height Z-scores
